# N-glycosylation Modification Reveals Insights into the Oxidative Reactions of Liver in Wuzhishan Pigs

**DOI:** 10.3390/molecules29225222

**Published:** 2024-11-05

**Authors:** Yuwei Ren, Feng Wang, Ruiping Sun, Yan Zhang, Xinli Zheng, Hailong Liu, Linlin Chen, Yanning Lin, Yujie Zhao, Mingxia Liang, Zhe Chao

**Affiliations:** Key Laboratory of Tropical Animal Breeding and Disease Research, Institute of Animal Science and Veterinary Medicine, Hainan Academy of Agricultural Sciences, Haikou 571100, China

**Keywords:** WZS pigs, proteomics, N-glycosylation, liver, oxidation

## Abstract

Although porcine liver contributes to their growth and development by nutrition production and energy supply, oxidative stress-induced hepatocyte damage is inevitable during metabolism. N-glycosylation is a common modification in oxidation; nevertheless, the effects of N-glycosylation on pig liver oxidative reactions remain undefined. In this study, liver proteins with N-glycosylation were detected in Wuzhishan (WZS) pigs between 4 and 8 months old and Large White (LW) pigs at 4 months old based on LC-MS/MS. The results showed that the number of differentially expressed proteins (DEPs) was larger between different pig cultivars than that between WZS pigs at various growth periods. The enriched pathways of DEPs were mainly related to oxidative reactions, and 10 proteins were finally selected that primarily consisted of CYPs, GSTs and HSPs with expressions significantly correlating to liver size and weight. The oxidative genes shared N-glycosylation-modified models of N-x-S and N-G. Five out of 10 proteins were upregulated in WZS pigs compared to LW pigs at 4 months old, while five proteins increased in WZS pigs from 4 to 8 months old. In conclusion, this research provides valuable information on the N-glycosylation motifs in liver oxidation genes of WZS pigs.

## 1. Introduction

The redox reaction in the liver tissue contributes to the growth and development of pigs by nutrition production and energy supply; however, this reaction releases reactive oxygen species (ROS) and inevitably brings oxidative stress, which further impairs hepatocytes and leads to liver diseases [1]. On the other hand, N-glycosylation is one category of common protein modification that affects biological processes such as cell adhesion, proliferation, differentiation, signal transduction, antioxidant ability, and inflammation [2]. Nevertheless, the effect of N-glycosylation on liver oxidative reactions have been little studied.

ROS could be produced by a series of components during metabolism processes and immune functions, such as proinflammation, lipid peroxidation, and drug catalysis, leading to oxidative stress and liver disease [3,4]. Glutathione-S-transferase (GST) and cytochrome P450 (CYP) activities are both responsible for antioxidant quality and detoxification functions in livestock liver [5,6]. An elevated abundance of GST proteins played an important role in regulating hepatic fatty acid oxidation [7], and the increasing mRNA expression of GST could enhance antioxidant ability and improve lipid metabolism in weaned piglets [8]. Moreover, CYP enzymes catalyzed a medicinal compound of scoparone and reduced oxidation reaction in liver [9], and CYP also participated in stearic acid catalysis, which are relevant oxidative reactions and detoxification processes [10]. In addition, both glutathione peroxidase (GSH-PX) activation and the mRNA expression level of HSPs increased in broilers under low temperature to enhance the antioxidant ability and alleviate cytochrome c-mediated apoptosis in hepatic tissue [11]. 

The key step of N-glycosylation is the co-translational transfer of the glycan on Asn to form the classic sequence of AsN-x-S er/Thr (where X represents any animo acid except Pro) in the nascent polypeptide chain [12]. N-glycosylation display widespread association with liver oxidative stress by impacting the stability of the antioxidant genes [13]. For instance, ROS production promotes normal protein structures switching from glycolytic metabolism to oxidative phosphorylation, increasing the N-glycosylated level of Mer tyrosine kinase in hepatocellular carcinoma [14]. The ammonia-induced activation of nitric oxide synthases or NADPH oxidase was related to an increasing N-glycosylation level of drug transport protein in human hepatic encephalopathy disease [15]. Thus, oxidative stress could increase N-glycosylation levels and further impair hepatocellular function.

WZS minipigs are native to Hainan Province in China, and characterized by tasty meat and strong resistance to a subtropical climate. The liver oxidative reaction is ubiquitous in metabolic processes and immunoreaction, and important for pig growth and development, but this process is accompanied by ROS production and oxidative stress; on the other hand, oxidative reaction is closely related to N-glycosylation. Nevertheless, the relationship between N-glycosylation and liver oxidative reactions is not clear; therefore, the aim of this study was to identify the connection of oxidation genes and N-glycosylation modification, and reveal the effects of oxidation genes with N-glycosylation on the liver oxidative reaction in WZS pigs.

## 2. Results

### 2.1. Proteome Analysis

**Figure 1:**
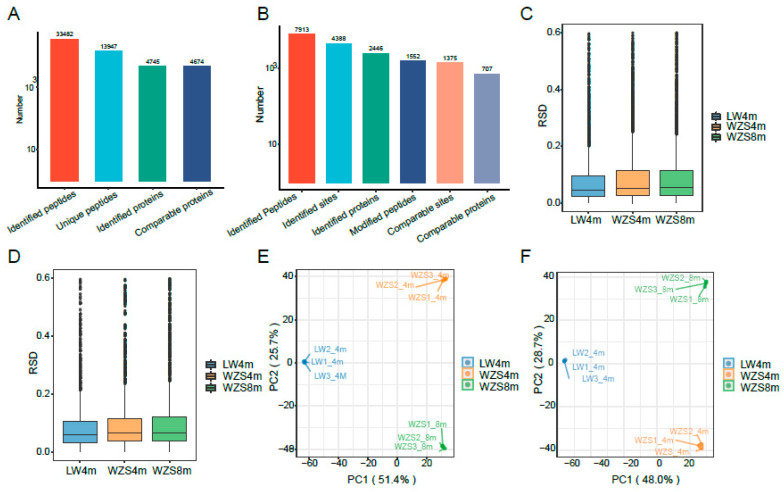
The number of total and modified peptides, sites, and proteins. (**A**) The number of total peptides and proteins, the comparable proteins identified by LC-MS/MS, and the comparable proteins indicated as all three biological duplications could be detected as expression signals. (**B**) The number of modified peptides, sites, and proteins with N-glycosylation, and the comparable sites indicated as all three biological duplications, could be detected as expression signals of both total proteins and modified sites, and then the comparable proteins were corresponding proteins to the comparable sites. The RSD analysis of total proteins (**C**) and modified proteins (**D**) in biological duplications of WZS pigs at 4 and 8 months old, and LW pigs at 4 months old. The PCA analysis of total proteins (**E**) and modified proteins (**F**) in biological duplications.

A total of 13,947 unique peptides and 4745 proteins were detected by LC-MS/MS (Figure 1A, Table S1), while 4388 sites, 7913 peptides, and 2446 proteins were identified with acetylation on the Met N-terminal (Figure 1B, Table S2). Most of the peptides were distributed at a length of 7–26 amino acids (Figure S1). In addition, 4674 proteins identified by LC-MS/MS were quantifiable and comparable (Figure 1A, Table S1), while 1375 sites and 707 proteins identified with N-glycosylation were quantifiable and comparable (Figure 1B, Table S2). The identified peptides were high-quality identified by three statistical approaches, as the relative standard deviation (RSD) of each group was determined to be less than 10% (Figure 1C,D), the principal component analysis (PCA) of each group held together (Figure 1E,F), and the Pearson’s correlation coefficient of samples in each group were strongly correlated (Figure S2).

### 2.2. Differentially Expressed Proteins (DEPs)

**Figure 2:**
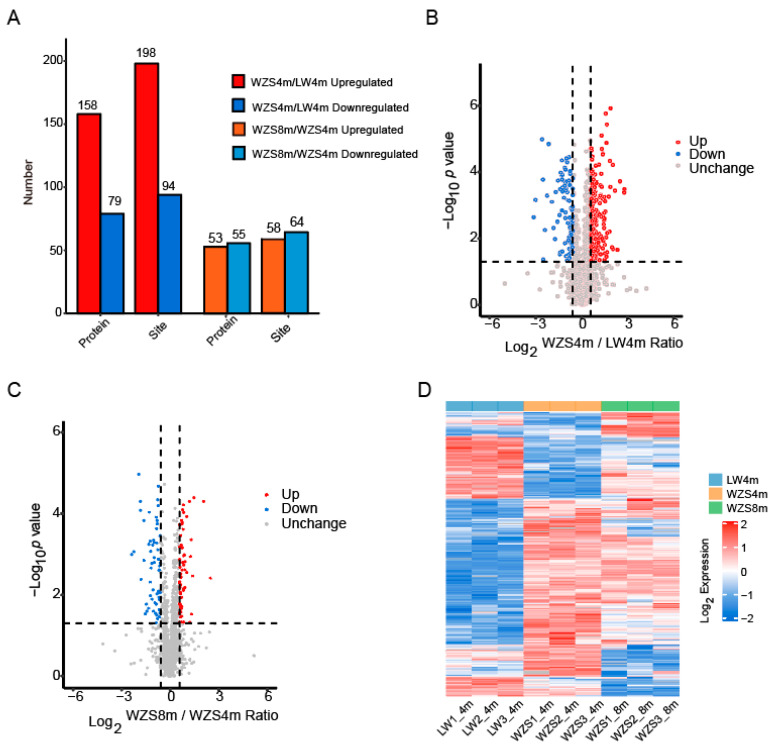
The expression of proteins in WZS and LW pigs. (**A**) The difference between numbers of DEPs in groups of WZS and LW pigs at 4 months old, and WZS pigs at 4 months old and 8 months old; comparison between the DEPs and unchanged expression proteins viewed by volcano plot of WZS and LW pigs at 4 months old (**B**), and WZS pigs at 4 months old and 8 months old (**C**), the dashed lines of horizontal axis presented as Log_2_^(WZS8m/WZS4m Ratio = 0.67/1.5)^, and the dashed lines of vertical axis presented as −Log_10_^(*p* value = 0.05)^; (**D**) the DEPs viewed by heatmap.

Different expression of proteins in WZS and LW pigs were analyzed to determine the differentially expressed proteins (DEPs). A number of 1140 proteins identified by LC-MS/MS contained 398 upregulated DEPs and 742 downregulated DEPs, and 238 proteins identified with N-glycosylation including 158 upregulated DEPs and 79 downregulated DEPs of WZS pigs at 4 months old compared to LW pigs at the same age according to the fold change ≤0.67 and ≥1.5 (*p* ≤ 0.05) (Figure 2A,B, Table S3). The 535 comparable proteins consisted of 266 upregulated DEPs and 269 downregulated DEPs, and 108 comparable proteins with N-glycosylation including 53 upregulated DEPs and 55 downregulated DEPs of WZS pigs at 8 months old compared to those at 4 months old with a fold change ≤0.67 and ≥1.5 (*p* ≤ 0.05) (Figure 2A,C, Table S4). Moreover, 252 DEPs were identified in common among WZS and LW pigs at 4 months old and WZS pigs at different growth periods. The comparison of DEPs suggested that protein expression showed more change between different pig cultivars than that in WZS pigs at various growth periods (Figure 2D).

### 2.3. Functional Analysis of DEPs

**Figure 3:**
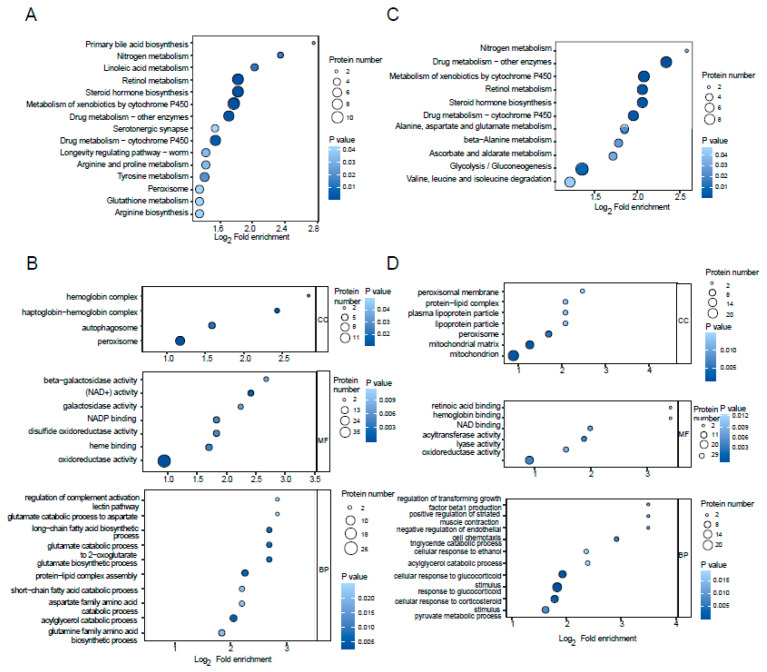
The functional enrichment of DEPs. Comparison between the KEGG (**A**) and GO (**B**) enrichment of DEPs in WZS and LW pigs at 4 months old and the KEGG (**C**) and (**D**) GO enrichment of DEPs of WZS pigs at 4 months old and 8 months old.

The 238 DEPs with N-glycosylation of WZS and LW pigs at 4 months old, and the 108 DEPswith N-glycosylation of WZS pigs at 4 months old and 8 months old, were analyzed via functional enrichment by searching the KEGG and GO databases. Among the enrichment functions, the analyzed pathways related to oxidation consisted of retinol metabolism, glutathione metabolism, tyrosine metabolism, and peroxisome from the KEGG database (Figure 3A), plus NADP binding, disulfide oxidoreductase, and short-chain fatty acid catabolic process, and zinc-binding dehydrogenase from protein domains from the GO terms of WZS and LW pigs at 4 months old (Figure 3B). However, several different pathways were enriched in WZS pigs at 4 months old and 8 months old, including alanine metabolism (Figure 3C), mitochondrion, response to glucocorticoid, and striated muscle contraction (Figure 3D), while cytochrome P450, glutathione S-transferase, retinol metabolism, and hemoglobin complex were collected in both compared groups.

### 2.4. Oxidation DEP Selection

Furthermore, an intersection protein set of 14 oxidation DEPs with N-glycosylation from the enriched pathways was selected and distributed to different categories of functions between both groups of WZS and LW pigs at 4 months old, and WZS pigs at 4 months old and 8 months old. They were cytochrome P450 subfamily genes of CYP2B6, CYP2D6, CYP1A2, and ubiquinol-cytochrome c reductase core protein 2 (UQCRC2), the glutathione S-transferase (GST) genes of GSTZ1, oxidases of peroxisomal 14 (PEX14) and metalloenzyme multicopper oxidase gene of coagulation factor V (F5), heat shock proteins (HSPs) of HSP90B1, HSPG2, and HSP70 (HSPA8), and the fatty acid metabolism gene of orosomucoid 1 (ORM1), free haptoglobin-mediated oxidative damage-related gene scavenger receptor CD163, and vitronectin (VTN) linked to myelin damage, as well as the glutamate metabolism gene of ionotropic glutamate delta receptors 1 (GLUD1). In addition, the expression of three CYPs, three HSPs, and GSTZ1 changed significantly, while CYP2D6, HSPA8, and GSTZ1 were downregulated in WZS pigs compared to LW pigs at 4 months old, and increased from 4 months old to 8 months old in WZS pigs, while other proteins, such as PEX14, ORM1, UQCRC2, and VTN exhibited a similar expression trend. GLUD1 expression was upregulated both in WZS pigs compared to LW pigs, and in WZS pigs at 8 months old compared to those 4 months old.

**Figure 4:**
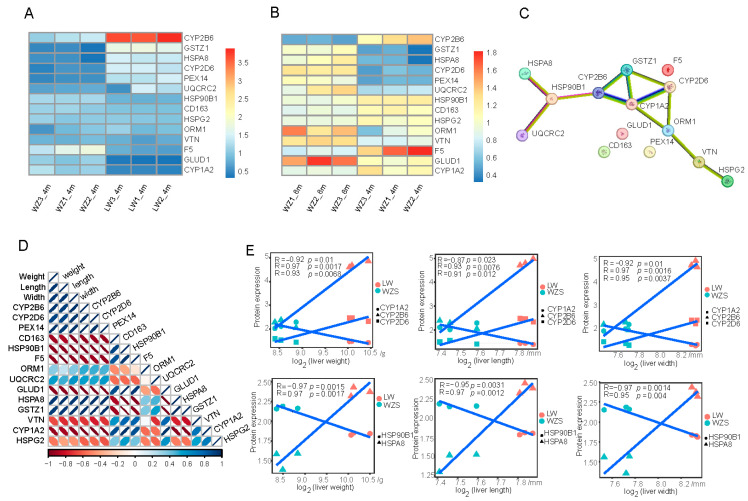
The expressions of oxidation-related proteins and their interactions. Comparison between the protein expression of 14 oxidative proteins in WZS and LW pigs at 4 months old (**A**), and WZS pigs at 4 months old and 8 months old (**B**); (**C**) the PPI network of 14 oxidative proteins; (**D**) the correlations inside and between the expressions of 14 protein and liver parameters; (**E**) the correlations between protein expressions of CYPs and HSPs and liver weight, length, and width.

The common oxidation DEPs were concentrated in the GST and CYP subfamilies, and these two protein clusters were connected closely with each other based on the protein–protein interaction (PPI) network. Specifically, CYP2B6 was linked with HSP90B1 and GSTZ1, and GSTZ1 was also connected with both CYP1A2 and CYP2D6 directly (Figure 4C), indicating that the CYP subfamily is a central system that mediates a series of oxidative activities. Furthermore, the Pearson’s correlation coefficient between protein expression and liver parameters showed that 10 out of 14 proteins were significantly correlated with liver weight, length, and width (Table S6), including CYPs (CYP2B6, CYP2D6, and CYP1A2) and HSPs (HSP90B1 and HSPA8) (Figure 4E). In addition, the expressions of proteins in the central line of PPI (HSPA8, HSP90B1, CYP2B6, CYP2D6, CYP1A2, and GSTZ1) were significantly correlated with each other (Figure 4D). Although ORM1 was linked directly to both CYP1A2 and CYP2D6 (Figure 4C), its expression was not significantly correlated with liver size and weight, suggesting ORM1 might affect liver function through other aspects but not liver size and weight. On the other hand, while CD163 and F5 were not in the central line of PPI, they were significantly correlated with liver size and weight, indicating they might participate in oxidation through connecting other proteins but not CYPs. As a result, 10 proteins were finally selected as oxidation proteins with N-glycosylation related to liver oxidation reactions in different pig cultivars. Five out of the ten oxidative proteins were upregulated in WZS pigs compared to LW pigs at 4 months old, while five proteins increased in WZS pigs from 4 months old to 8 months old.

### 2.5. The Oxidation DEPs’ Domains and Motifs

**Figure 5:**
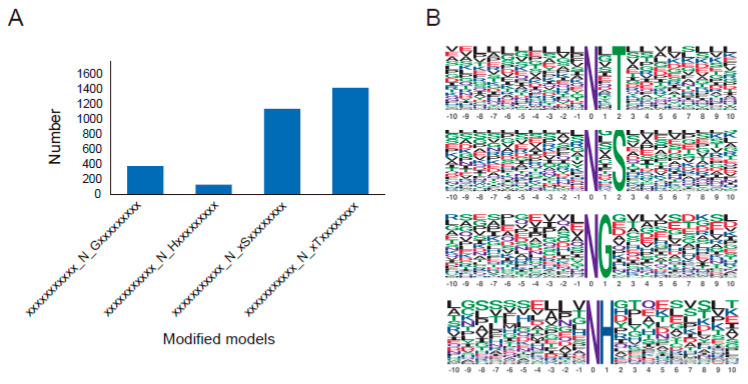
The motif of identified proteins with N-glycosylation. (**A**) The four categories of motifs; (**B**) the modified patterns of motifs.

A total of 3078 modified models were obtained and divided into four motifs (Figure 5A). The most common motifs were N-glycosylation sequences that accounted for 78.3%, which weref N-x-T (43.5%) and N-x-S (34.8%), followed by the N-G motif (11.5%) (Figure 5B) N-glycosylation. The 10 selected oxidation DEPs presented four categories of motif; PEX14 showed N-x-T, five DEPs shared the motif of N-x-S, and three DEPs exhibited the motif of N-G (Table 1), suggesting the two motifs might be the major patterns in the oxidation reactions. 

## 3. Discussion

This study compared the linkage of liver N-glycosylation modification and oxidation in groups of WZS and LW pigs at 4 months old, and WZS pigs with different growth periods of 4 months and 8 months. By comparing the number of DEPs, analyzing the functional enrichment of DEPs based on various databases, calculating the Pearson’s correlation coefficient for the expression of proteins selected from oxidative pathways and the liver parameters, and the screening of modified models in different pig groups, we finally selected 10 proteins and their important motifs related to oxidation. The number of upregulated proteins identified with N-glycosylation was more than that of the downregulated proteins in WZS compared to LW pigs, while it was less in WZS pigs 8 months old than in those 4 months old. The DEPs showed more volatility between different pig cultivars at the same age than in WZS pigs of various growth periods. In addition, most of the enrichment functions were associated with oxidation reactions, such as the broad-spectrum terms of xenobiotic metabolism by cytochrome P450, retinol metabolism, and glutathione metabolism.

Furthermore, the 10 selected oxidation proteins with N-glycosylation consisted of three CYPs, one GST, and two HSPs, indicating the three subfamilies of CYP, GST, and HSP were the primary members with N-glycosylation modifications due to oxidation reactions. In this study, CYP functioned as a central system linked with GST and HSP. CYP enzymes are a category of heme-thiolate monooxygenases that play a primary role in catalyzing the oxidation of aliphatic and aromatic C-H bonds [16,17], and also contribute to the metabolism of xenobiotics in human liver [18]. CYP1A2 and CYP2B6 participated in drug metabolism and regulated oxidation reactions in hepatocytes [19]. CYP2D6 contributed to metabolize a variety of substrates, such as lipids and drugs, for the purpose of liver energy supply, anti-inflammation, and antioxidation [20]. Moreover, GST is known as a detoxification enzyme system that protects against the pathoproteins of various liver diseases and oxidative stress [21]. The protein expression values of GSTs were upregulated at higher altitude compared to lower altitude to enhance antioxidative capacity in Tibetan pig livers [22], which was similar to the increase of GST expression in the subtropics for detoxification in WZS pig livers in this study. The depletion of GSTZ1 causes oxidative stress and detoxification in the liver, leading to the disorder of energy metabolism [23]. The protein expression of HSP90B1 is closely related to the oxidative stress caused by ROS production [24]; GLUD1 is a critical enzyme participating in glutamine metabolism, and could inhibit hepatocellular carcinoma progression through regulating the ROS production and oxidative stress state in mitochondria [25]. CD163 prevented hepatobiliary injury in sickle cell disease by means of inhibiting oxidative stress, inflammation, and thrombosis [26,27]; and the reducing PEX14 impaired peroxisomes and caused liver oxidative stress [28]. In addition, the selected oxidation proteins were closely connected with each other. GSTZ1 was conjoined to three CYPs (CYP1A2, CYP2B6, and CYP2D6) on the central line in the PPI network, and CYP2B6 was linked to HSP90B1, indicating the CYP subfamily could interact with GST, HSP, and other proteins to regulate oxidation reactions. Despite the connection of ORM1 to CYP2D6 and CYP1A2 in the PPI of this study, the expression of ORM1 was not significantly correlated with liver size and weight, suggesting ORM1 might influence liver function through other aspects but not liver size and weight. 

The expression level of oxidation proteins might be varied under different conditions. For example, the mRNA expression of CYP1A2 and CYP2B6 was enhanced during the drug metabolism in hepatocytes compared to cells without drug treatment [19], and CYP2D6 overexpression impaired liver by increasing pro-inflammatory cytokines’ expression level and inducing hepatic oxidative stress [29]; however, CYP2B6 and CYP2D6 were downregulated and CYP1A2 were upregulated as N-glycosylation DEPs in WZS pigs compared to LW pigs at 4 months old, while CYP2B6 and CYP1A2 were downregulated and CYP2D6 was upregulated in WZS pigs at 8 months old compared to those 4 months old. In addition, GSTZ1 gradually increased during adulthood of rats to regulate antioxidative stress with aging [30]; although GSTZ1 was downregulated in WZS pigs compared to LW pigs at 4 months old, it was upregulated in WZS pigs at 8 months old compared to those 4 months old. As a result, five oxidation proteins with N-glycosylation related to liver size and weight were upregulated during the peak growth period of WZS pigs at 4 months old, namely CD163, HSP90B1, F5, GLUD1, and CYP1A, while five proteins exhibited a sustainable growth trend in WZS pigs from 4 months old to 8 months old, namely CYP2D6, PEX14, GLUD1, HSPA8, and GSTZ.

The N-glycosylation model analysis demonstrated that the top two motifs were N-x-T (43.5%) and N-x-S (34.8%), and the third was the N-G pattern (11.5%). Notably, the N-x-T was identified in PEX14, while the N-x-S and N-G patterns were detected in five DEPs and three DEPs, separately. The proteins of CD163, HSP90B1, F5, GSTZ1, and CYP2B6 shared the N-x-S pattern, while CYP2D6, GLUD1, and HSPA8 exhibited the N-G pattern. Early in the year of 2010, human IgG1 and IgG2 were found in non-classical N-glycosylation sites on Gln [31], and human IgG was detected less commonly on N-glycosylation sites in epithelial cancer cells than in healthy cells [32]. Similarly, the N-glycosylation of the N-G pattern in oxidation proteins might indicate different redox states under oxidative stress in this study, though the N-G pattern function requires further in-depth investigation.

## 4. Materials and Methods

### 4.1. Sample Collection

The 6 WZS pigs, a 4-month-old castrated boar and 2 sows, and an 8-month-old castrated boar and 2 sows, were purchased from the Wuzhishan Preservation Factory (Chengmai city, China), and 3 Large Whites (LWs), 1 boar and 2 sows 4 months old, were purchased from the Nongkenhongmu Agricultural Development Company (Qiongzhong city, China). These pigs were fed in a similar environment with free food and water for an adaptive week before slaughtering. After one week, all the pigs had food withdrawn for 24 h, and were slaughtered in the HAAS Yongfa pig facility. The pigs were stunned via electric shock, exsanguinated, and dissected without consciousness. The livers were measured to record weight, length, and width (Table S7), and samples were collected from the middle part of the livers’ right lateral lobe. All the samples were frozen directly in liquid nitrogen and transferred to the library and stored at −80 °C before protein extraction.

### 4.2. Peptide Preparation

The samples were taken from the −80 °C freezer, put into a mortar pre-cooled with liquid nitrogen, fully ground to powder, and total protein was extracted using a kit (Thermo Scientific T-PER, Cat. No. 78,510, Rockford, IL, USA), and the protein concentration was determined using the Pierce^TM^ BCA protein assay kit (Thermo Scientific Cat. No. 23,227, Rockford, IL, USA, 10 × 1 mL glass ampules). The protein lysate of each sample was adjusted to an equal quantity after trypsin digestion. The peptide was dissolved in 200 μL enriched buffer solution (80% acetonitrile, 5% trifluoroacetic acid), and supernatant was transferred to a column (hydrophilic action liquid chromatography, HILIC) [33], and centrifuged for 15 min at 1000 g/min, eluted for 3 times by enriched buffer solution, and glycopeptides were collected by eluting with 0.1% trifluoroacetic acid, 50 mM ammonium bicarbonate solution, and 50% acetonitrile, respectively. The glycopeptides were dried in the Speedvac (Thermo Scientific SRF110P1-115, Asheville, NC, USA) and redissolved in 50 mM ammonium bicarbonate within heavy oxygen water, before addition of 2 μL PNGase F glycosidase to digest at 37 °C overnight, and desalted for liquid chromatography–mass spectrometry (LC-MS/MS) analysis in Jingjie PTM BioLab Co., Ltd., Hangzhou, China [34].

### 4.3. LC-MS/MS Analysis

The peptides were dissolved in solvent A (0.1% formic acid, 2% acetonitrile water solution), then loaded into a reversed-phase analytical column (25-cm length, 100 μm i.d.). The mobile phase contained solvent A and solvent B (0.1% formic acid, 90% acetonitrile water solution). Peptides were separated with the gradient setting: 0–18.5 min, 7–24% B; 18.5–25 min, 24–35% B; 25–28 min, 35–80% B; 28–30 min, 80% B, and flow rate maintained at 700 nL/min on an EASY-nLC 1200 UPLC system (Thermo Scientific). The separated peptides were ionized and analyzed with an Orbitrap Exploris 480 (Thermo Scientific Cat. No BRE725539, San Jose, CA, USA) in a nano-electrospray ion environment. The electrospray voltage was set at 2300 V, and the FAIMS compensated voltage (CV) was −45 V. Both precursors and fragments were detected and analyzed using the Orbitrap Exploris 480 (Thermo Scientific Cat. No BRE725539, San Jose, CA, USA). The full MS scan resolution was set at 120,000, and the secondary mass spectroscopy scan was fixed with starting mass at 120.0 *m*/*z* and with a resolution of 45,000. Data collection was performed using data-independent acquisition (DIA) [35] technology. Automatic gain control (AGC) was set at 1 × 10^6^, with maximum injection time of automatic, to enhance the efficiency of mass spectrometry.

### 4.4. Database Search

The DIA data were processed using Spectronaut (v.17.0) software with a built-in Pulsar search engine under software false parameters to construct the spectral library. Tandem mass spectra were searched according to Sus_scrofa_9823_PR_20230529.fasta (46,179 entries, https://www.uniprot.org/proteomes/UP000008227, accessed on 5 May 2024) database, and the searched data are available via ProteomeXchange [36] with identifier PXD056683. The enzyme cutting model was set as trypsin/P, with the max missing cleavages at 2. Fixed modification was specified as carbamidomethyl on Cys, while variable modifications were specified as acetylation on protein N-terminal, oxidation on methionine (Met) and N-glycosylation. The false discovery rate (FDR) of proteins, peptides, and the peptide-spectrum matches (PSM) was adjusted to <1%. The searching library was further filtered under two conditions, with precursor and protein FDR settings at 1%, and the identified proteins contained at least one unique peptide. The localization probability of all the identified peptides and proteins were adjusted to >0.75 [37]. The identified peptides were quantified and their statistical consistency tested among biological duplication samples. Three parameters of Pearson’s correlation coefficient, principal component analysis (PCA), and relative standard deviation (RSD) were used to analyze the data consistency [38]. 

The modified models were calculated as 10 bases upstream and downstream away from the candidate N-glycosylation site, when the number of peptide segments in the form of a feature sequence was more than 20 and *p* value ≤ 0.000001. The feature sequence was considered as a motif of modified peptides.

### 4.5. Functional Analysis

Differentially expressed proteins (DEPs) were compared from groups of WZS and LW pigs at 4 months old, and WZS pigs at 4 months old and 8 months old, with the requirement of fold enrichment (FC) ≥ 1.2 and *p* value ≤ 0.05 using Student’s *t* test. The DEPs were functionally enriched based on KEGG and GO databases, and Fisher’s exact test was used to analyze the significance of functional enrichment of DEPs, taking the identified protein as the background, and the enriched functional terms with fold enrichment ≥ 1.5 and *p* value ≤ 0.05 were considered as significant.

The protein–protein interaction (PPI) network of oxidation proteins was searched against the STRING database. STRING uses a confidence score to detect the interaction of proteins in the network, and a confidence score ≥ 0.7 was considered as high confidence.

### 4.6. Oxidation Proteins Selection

The intersection protein set was selected from enrichment pathways of DEPs in WZS and LW pigs at 4 months old and WZS pigs at different growth periods. Pearson’s correlation coefficient [39] analysis was used to compare the protein expression and liver parameters of weight, length, and width, and the reserved proteins required the coefficient R ≥ 0.85 or R ≤ −0.85 with *p* value ≤ 005.

## 5. Conclusions

In summary, a total number of 238 DEPs and 108 DEPs with N-glycosylation were selected from groups of different pig cultivars the same age and WZS pigs at different growth periods, separately, and a number of 10 oxidation proteins was selected through the intersection of enriched pathways, and Pearson’s correlation coefficient analysis. The CYP subfamily might function as a central system to link GSTs, HSPs, and other proteins. Moreover, five oxidation proteins were upregulated in WZS pigs compared to LW pigs at 4 months old, namely CD163, HSP90B1, F5, GLUD1, and CYP1A, while five proteins displayed sustainable growth trend in WZS pigs from 4 months old to 8 months old, namely were CYP2D6, PEX14, GLUD1, HSPA8, and GSTZ1. In addition, the modified models of the DEPs with N-glycosylation were divided into four categories of motifs, and the classic models accounted for 78.3% (N-x-T was 43.5% and N-x-S was 34.8%). Among the 10 selected oxidation proteins, PEX14 showed N-x-T, five DEPs displayed N-x-S, and three DEPs exhibited N-G, suggesting the motifs of N-x-S and N-G might be the most common models in oxidation reactions. Although this research has certain limitations, it provides important information on liver N-glycosylation modification in WZS and LW pigs at the same growth period, and WZS pigs at different growth periods, which contributes to elucidate the molecular mechanisms of oxidative reactions in the liver of WZS pig.

## Figures and Tables

**Table 1:** The modified sequences of the oxidation proteins.

Protein Name	Modified Sequence	Motif
CYP2B6	_SQGALQDPTYYFHSSTAN[+O-H-N]CSIVFGK_	xxxxxxxxxx_N_xSxxxxxxxx
CYP2D6	_PVVVLN[+O-H-N]GLAAVR_	xxxxxxxxxx_N_Gxxxxxxxxx
PEX14	_ASSEQAEQPSQPN[+O-H-N]STPGSENVVPR_	xxxxxxxxxx_N_xTxxxxxxxx
CD163	_ATGWAN[+O-H-N]FSAGSGR_	xxxxxxxxxx_N_xSxxxxxxxx
HSP90B1	_EEEAIQLDGLN[+O-H-N]ASQIR_	xxxxxxxxxx_N_xSxxxxxxxx
F5	_HISQDN[+O-H-N]SSSSSIGPLEDLSSDLLLLER_	xxxxxxxxxx_N_xSxxxxxxxx
GLUD1	_IIAEGAN[+O-H-N]GPTTPEADK_	xxxxxxxxxx_N_Gxxxxxxxxx
HSPA8	_LLQDFFN[+O-H-N]GK_	xxxxxxxxxx_N_Gxxxxxxxxx
GSTZ1	_TISRIN[+O-H-N]KSLLALEAFQVSH_	xxxxxxxxxx_N_xSxxxxxxxx
CYP1A2	_HVLVNQWQVN[+O-H-N]HDPK_	xxxxxxxxxx_N_Hxxxxxxxxx

N-glycosylation in the Modified sequence and motif meant sequences containing N-glycosylation sites.

## Data Availability

The mass spectrometry proteomics data have been deposited to the ProteomeXchange with identifier PXD056683.

## Supplementary Materials

The following supporting information can be downloaded at https://www.mdpi.com/article/10.3390/molecules29225222/s1, Figure S1: The distribution of peptides length; Figure S2: Pearson’s correlation coefficient of samples in WZS and LW pigs; Table S1: LC-MS/MS spectrum database search analysis summary; Table S2: Spectrum database search of acetylation on Met N-terminal analysis summary (localization probability > 0.75); Table S3: Differentially expressed protein summary; Table S4: Differentially modified sites (modified proteins) summary; Table S5: The expression of oxidation DEPs in WZS and LW pigs; Table S6: The correlation between the expression of oxidation proteins and liver parameters; Table S7: The parameters of pig liver.

## References

[1] Wada Y., Takeda Y., Kuwahata M. (2017). Potential role of amino acid/protein nutrition and exercise in serum albumin redox state. Nutrients.

[2] Schjoldager K.T., Narimatsu Y., Joshi H.J., Clausen H. (2020). Global view of human protein glycosylation pathways and functions. Nat. Rev. Mol. Cell Biol..

[3] Radovani B., Gudelj I. (2022). N-glycosylation and inflammation; the not-so-sweet relation. Front. Immunol..

[4] Farzaei M.H., Zobeiri M., Parvizi F., El-Senduny F.F., Marmouzi I., Coy-Barrera E., Naseri R., Nabavi S.M., Rahimi R., Abdollahi M. (2018). Curcumin in liver diseases: A systematic review of the cellular mechanisms of oxidative stress and clinical perspective. Nutrients.

[5] Sun J., Jia L., Chen X. (2023). Efficient adsorption and extraction of glutathione s-transferases with glutathione-functionalized graphene oxide-polyhedral oligomeric silsesquioxane composite. Molecules.

[6] Deng J., Zhao L., Zhang N.Y., Karrow N.A., Krumm C.S., Qi D.S., Sun L.H. (2018). Aflatoxin B_1_ metabolism: Regulation by phase I and II metabolizing enzymes and chemoprotective agents. Mutat. Res. Rev. Mutat. Res..

[7] Ribeiro D.M., Leclercqc C.C., Charton S.A.B., Costa M.M., Carvalho D.F.P., Sergeant K., Cocco E., Renaut J., Freire J.P.B., Prates J.A.M. (2024). The impact of dietary Laminaria digitata and alginate lyase supplementation on the weaned piglet liver: A comprehensive proteomics and metabolomics approach. J. Proteom..

[8] Xu X., Chen X., Huang Z., Chen D., Yu B., Chen H., He J., Luo Y., Zheng P., Yu J. (2019). Dietary apple polyphenols supplementation enhances antioxidant capacity and improves lipid metabolism in weaned piglets. J. Anim. Physiol. Anim. Nutr..

[9] Fayyaz A., Makwinja S., Auriola S., Raunio H., Juvonen R.O. (2018). Comparison of in vitro hepatic scoparone 7-o-demethylation between humans and experimental animals. Planta Med..

[10] Esposito S., Krick A., Pasquier O., Bonche F., Ingenito R., Magotti P., Bianchi E., Monteagudo E., Gallo M., Cicero D.O. (2023). Fatty acid acylated peptide therapeutics: Discovery of omega-n oxidation of the lipid chain as a novel metabolic pathway in preclinical species. J. Pharm. Biomed. Anal..

[11] Lyu Q., Feng M., Wang L., Yang J., Wu G., Liu M., Feng Y., Lin S., Yang Q., Hu J. (2022). Taurine prevents liver injury by reducing oxidative stress and cytochrome c-mediated apoptosis in broilers under low temperature. Adv. Exp. Med. Biol..

[12] Esmail S., Manolson M.F. (2021). Advances in understanding N-glycosylation structure.; function.; and regulation in health and disease. Eur. J. Cell Biol..

[13] Aldonza M.B.D., Cha J., Yong I., Ku J., Sinitcyn P., Le D., Cho R.E., Delos Reyes R.D., Kim D., Kim S. (2023). Multi-targeted therapy resistance via drug-induced secretome fucosylation. eLife.

[14] Liu Y., Lan L., Li Y., Lu J., He L., Deng Y., Fei M., Lu J.W., Shangguan F., Lu J.P. (2022). N-glycosylation stabilizes MerTK and promotes hepatocellular carcinoma tumor growth. Redox Biol..

[15] Jördens M.S., Keitel V., Karababa A., Zemtsova I., Bronger H., Häussinger D., Görg B. (2015). Multidrug resistance-associated protein 4 expression in ammonia-treated cultured rat astrocytes and cerebral cortex of cirrhotic patients with hepatic encephalopathy. Glia.

[16] Coleman T., Podgorski M.N., Doyle M.L., Scaffidi-Muta J.M., Campbell E.C., Bruning J.B., De Voss J.J., Bell S.G. (2023). Cytochrome P450-catalyzed oxidation of halogen-containing substrates. J. Inorg. Biochem..

[17] Tang X., Xiong K., Li M. (2023). Effects of dietary epidermal growth factor supplementation on liver antioxidant capacity of piglets with intrauterine growth retardation. J. Anim. Sci..

[18] Uehara S., Yoneda N., Higuchi Y., Yamazaki H., Suemizu H. (2022). Cytochrome P450-dependent drug oxidation activities and their expression levels in liver microsomes of chimeric TK-NOG mice with humanized livers. Drug Metab. Pharmacok..

[19] Bachour-El Azzi P., Chesné C., Uehara S. (2022). Expression and functional activity of cytochrome P450 enzymes in human hepatocytes with sustainable reproducibility for in vitro phenotyping studies. Adv. Pharmacol..

[20] Ma Z., Liang H., Wang S., Miao W., Yu L., Liu S., Luo Z., Su S., Wang J., Liu S. (2024). Nardosinone relieves metabolic-associated fatty liver disease and promotes energy metabolism through targeting CYP2D6. Phytomedicine.

[21] Prysyazhnyuk V., Sydorchuk L., Sydorchuk R., Prysiazhniuk I., Bobkovych K., Buzdugan I., Dzuryak V., Prysyazhnyuk P. (2021). Glutathione-S-transferases genes-promising predictors of hepatic dysfunction. World J. Hepatol..

[22] Gu X., Chang X., Yang L., Chamba Y., Geng F. (2023). Quantitative proteomic analysis of Tibetan pig livers at different altitudes. Molecules.

[23] Squirewell E.J., Mareus R., Horne L.P., Stacpoole P.W., James M.O. (2020). Exposure of rats to multiple oral doses of dichloroacetate results in upregulation of hepatic glutathione transferases and NAD(P)H dehydrogenase [Quinone] 1. Drug Metab. Dispos..

[24] Xu M., Qi Q., Men L., Wang S., Li M., Xiao M., Chen X., Wang S., Wang G., Jia H. (2020). Berberine protects Kawasaki disease-induced human coronary artery endothelial cells dysfunction by inhibiting of oxidative and endoplasmic reticulum stress. Vascul. Pharmacol..

[25] Zhao Q., Yu M., Li J., Guo Y., Wang Z., Hu K., Xu F., Liu Y., Li L., Wan D. (2024). GLUD1 inhibits hepatocellular carcinoma progression via ROS-mediated p38/JNK MAPK pathway activation and mitochondrial apoptosis. Discov. Oncol..

[26] Liu S., Zhang C., Maimela N.R., Yang L., Zhang Z., Ping Y., Huang L., Zhang Y. (2019). Molecular and clinical characterization of CD163 expression via large-scale analysis in glioma. Oncoimmunology.

[27] Kaminski T.W., Sivanantham A., Mozhenkova A., Smith A., Ungalara R., Dubey R.K., Shrestha B., Hanway C., Katoch O., Tejero J. (2024). Hemoglobin scavenger receptor CD163 as a potential biomarker of hemolysis-induced hepatobiliary injury in sickle cell disease. Am. J. Physiol. Cell Physiol..

[28] Guo Y., Zhou P., Qiao L., Guan H., Gou J., Liu X. (2023). Maternal protein deficiency impairs peroxisome biogenesis and leads to oxidative stress and ferroptosis in liver of fetal growth restriction offspring. J. Nutr. Biochem..

[29] Lei Y., Chen Y., Wang S., Lin Z., Han P., Tian D., Wang H., Liu M. (2024). L-lysine supplementation attenuates experimental autoimmune hepatitis in a chronic murine model. Exp. Anim..

[30] Xu S., Hou D., Liu J., Ji L. (2018). Age-associated changes in GSH S-transferase gene/proteins in livers of rats. Redox Rep..

[31] Valliere-Douglass J.F., Eakin C.M., Wallace A., Ketchem R.R., Wang W., Treuheit M.J., Balland A. (2010). Glutamine-linked and non-consensus asparagine-linked oligosaccharides present in human recombinant antibodies define novel protein glycosylation motifs. J. Biol. Chem..

[32] Huang X., Zhang S., Tang J., Tian T., Pan Y., Wu L., Zhang J., Liu Y., Huang J., Dai H. (2023). A self-propagating c-met-sox2 axis drives cancer-derived igg signaling that promotes lung cancer cell stemness. Cancer Res..

[33] Buszewski B., Noga S. (2012). Hydrophilic interaction liquid chromatography (HILIC)—A powerful separation technique. Anal. Bioanal. Chem..

[34] Dolores-Hernández M., Morales-Hipólito E.A., Villaseñor A., López-Arellano R. (2022). Determination of zilpaterol in a residue depletion study using LC-MS/MS in cattle plasma.; muscle.; liver and kidney. Food Chem..

[35] Tsugawa H., Cajka T., Kind T., Ma Y., Higgins B., Ikeda K., Kanazawa M., VanderGheynst J., Fiehn O., Arita M. (2015). MS-DIAL: Data-independent MS/MS deconvolution for comprehensive metabolome analysis. Nat. Methods.

[36] Deutsch E.W., Bandeira N., Perez-Riverol Y., Sharma V., Carver J.J., Mendoza L., Kundu D.J., Wang S., Bandla C., Kamatchinathan S. (2023). The ProteomeXchange consortium at 10 years: 2023 update. Nucleic Acids Res..

[37] Krug K., Mertins P., Zhang B., Hornbeck P., Raju R., Ahmad R., Szucs M., Mundt F., Forestier D., Jane-Valbuena J. (2019). A curated resource for phosphosite-specific signature analysis. Mol. Cell. Proteom..

[38] Castellani F., Antonucci A., Pindinello I., Protano C., Vitali M. (2022). Determination of carbonyl compounds in different work environments: Comparison between LC-UV/DAD and LC-MS/MS detection methods. Int. J. Environ. Res. Public Health.

[39] Akoglu H. (2018). User’s guide to correlation coefficients. Turk. J. Emerg. Med..

