# Supplementary material for: N-glycosylation Modification Reveals Insights into the Oxidative Reactions of Liver in Wuzhishan Pigs
Source: Molecules. 2024 Nov 5;29(22):5222. doi: 10.3390/molecules29225222 (PMC11596063; doi:10.3390/molecules29225222)
Supplement: Supplementary file 1 [file molecules-29-05222-s001.zip › molecules-3281536-supplementary.pdf]

Supplemental tables and figures

Supplemental Figures

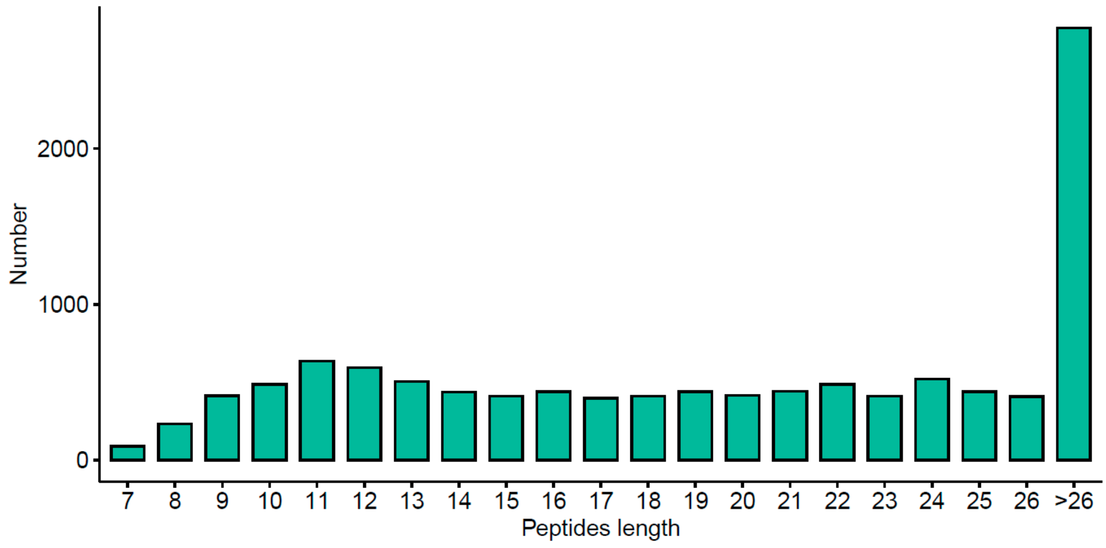

Figure S1 The distribution of peptides length

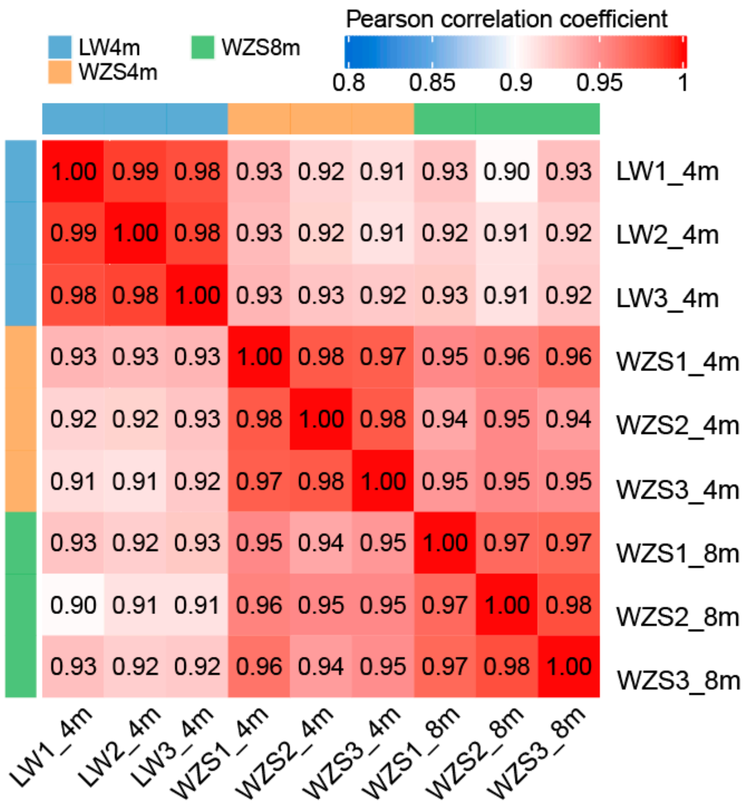

Figure S2 Pearson's correlation coefficient of samples in WZS and LW pig

Supplemental Tables

Table S1 LC-MS/MS spectrum database search analysis summary

Identified peptides	Unique peptides	Identified proteins	Comparable proteins
33482	13947	4745	4674

Table S2 Spectrum database search of acetylation on Met N-terminal analysis summary (Localization probability >0.75)

Peptides	Modified peptides	Identified proteins	Identified sites	Comparable proteins	Comparable sites
7913	1552	2446	4388	707	1375

Note: Peptides: number of peptides which spectrum hit; Modified peptides: total number of modified peptides identified; Identified proteins: number of proteins detected by spectrum search analysis; Identified sites: number of modification sites detected by spectrum search analysis; Comparable proteins: number of proteins comparable with both identified total and modified protein; Comparable sites: number of modification sites comparable.

Table S3 Differentially expressed protein summary

Compare group	Regulated type	fold change >1.2	fold change >1.3	fold change >1.5	fold change >2
WZSliv4m/LWliv4m	Up-regulated	774	615	398	179
	Down-regulated	1574	1219	742	336
WZSliv8m/WZSliv4m	Up-regulated	641	485	266	87
	Down-regulated	675	492	269	104

Table S4 Differentially modified sites (modified proteins) summary

Compare group	Regulated type	fold change >1.2	fold change >1.3	fold change >1.5	fold change >2
WZS8m/WZS4m	Up-regulated	122 (103)	92 (81)	58 (53)	25 (25)
	Down-regulated	182 (141)	123 (101)	64 (55)	11 (11)
WZS4m/LW4m	Up-regulated	397 (278)	329 (241)	198 (158)	67 (59)
	Down-regulated	150 (124)	129 (109)	94 (79)	44 (39)

Table S5 The expression of oxidation DEPs in WZS and LW pigs

Gene	LW1_4m	LW2_4m	LW3_4m	WZS1_4m	WZS2_4m	WZS3_4m	WZS1_8m	WZS2_8m	WZS3_8m	WZS8m/WZS4m Ratio	WZS8m/WZS4m P value	WZS4m/LW4m Ratio	WZS4m/LW4m P value
CYP2B6	3.618	3.876	3.695	1.313	1.404	1.080	0.504	0.537	0.481	0.401	0.0004	0.339	0.0001
CYP2D6	1.472	1.333	1.470	0.602	0.539	0.458	1.278	1.210	1.232	2.326	0.0004	0.374	0.0003
PEX14	1.302	1.459	1.305	0.551	0.541	0.470	1.091	1.025	0.942	1.957	0.0005	0.384	0.0001
CD163	0.950	0.958	0.961	1.157	1.059	1.104	0.861	0.955	0.899	0.818	0.0068	1.157	0.0048
HSP90B1	0.802	0.821	0.831	1.172	1.208	1.179	0.957	0.904	0.999	0.804	0.0019	1.450	1.11E-05
F5	0.685	0.666	0.813	1.662	1.810	1.338	0.791	0.876	0.824	0.518	0.002	2.221	0.0019
ORM1	0.878	0.952	1.103	0.888	1.027	0.587	1.596	1.244	1.421	1.703	0.0383	0.852	0.0371
UQCRC2	1.466	1.252	0.565	0.722	0.779	0.705	0.906	1.235	1.342	1.578	0.0229	0.633	0.0229
GLUD1	0.346	0.339	0.361	0.984	1.122	1.069	1.494	1.778	1.622	1.541	0.0024	3.032	1.33E-05
HSPA8	1.355	1.418	1.483	0.538	0.319	0.547	0.964	1.065	1.203	2.301	0.0103	0.330	0.0031
GSTZ1	1.779	1.655	1.717	0.520	0.322	0.483	1.017	0.975	1.082	2.318	0.0047	0.257	0.0007
VTN	0.672	0.773	0.790	0.813	0.905	0.835	1.252	1.334	1.316	1.529	0.0003	0.654	0.0003
CYP1A2	0.483	0.438	0.487	1.034	1.121	1.297	0.897	0.942	0.893	0.791	0.0283	2.449	0.0002
HSPG2	1.001	0.977	1.040	0.999	1.054	1.113	0.922	0.929	0.951	0.885	0.0206	1.048	0.2632

Table S6 The correlation between the expression of oxidation proteins and liver parameters

Proteins	cor_weight	*P*_weight	cor_length	*P*_length	cor_width	*P*_width
CYP2B6	0.964	0.002	0.937	0.006	0.976	0.001
CYP2D6	0.914	0.011	0.917	0.010	0.961	0.002
PEX14	0.977	0.001	0.945	0.004	0.971	0.001
CD163	-0.902	0.014	-0.871	0.024	-0.970	0.001
HSP90B1	-0.953	0.003	-0.959	0.002	-0.980	0.001
F5	-0.947	0.004	-0.990	<0.001	-0.956	0.003
ORM1	0.351	0.495	0.217	0.679	0.370	0.470
UQCRC2	0.565	0.242	0.500	0.313	0.590	0.218
GLUD1	-0.952	0.003	-0.954	0.003	-0.964	0.002
HSPA8	0.951	0.004	0.975	0.001	0.958	0.003
GSTZ1	0.940	0.005	0.958	0.003	0.972	0.001
VTN	-0.658	0.156	-0.733	0.097	-0.730	0.100
CYP1A2	-0.925	0.008	-0.886	0.019	-0.931	0.007
HSPG2	-0.537	0.272	-0.404	0.427	-0.450	0.370

Note: In this table, cor_weight, cor_length and cor_width presented the correlations between protein expressions and weight, length and width, separately; *P*_weight, *P*_length and *P*_width presented

the *P* value of correlations between protein expressions and weight, length and width, separately.

Table S7 The parameters of pig liver

Samples	Liverweight /g	Liverlength /mm	Liverwidth /mm
WZS4m1	338.5	176.4	184.04
WZS4m2	371.7	170.4	210.5
WZS4m3	466.4	193.24	214.82
LW4m1	1045.5	223.8	326.0
LW4m2	1392.6	234.0	323.0
LW4m3	1086.4	227.8	314.8
